# Attention-Deficit/Hyperactivity Disorder in Looked-After Children: a Systematic Review of the Literature

**DOI:** 10.1007/s40474-017-0116-z

**Published:** 2017-07-18

**Authors:** Renece Willis, Suyog Dhakras, Samuele Cortese

**Affiliations:** 1 Academic Unit of Psychology, University of Southampton, Southampton, UK; 2 Solent NHS Trust, Southampton, UK; 3 Clinical and Experimental Sciences (CNS and Psychiatry), Faculty of Medicine, University of Southampton, Southampton, UK; 4 The Child Study Center, New York University Langone Medical Center, New York, NY 10016 USA

**Keywords:** ADHD, Looked-after children, Psychostimulants

## Abstract

**Purpose of review:**

To systematically review the literature on the prevalence and pharmacological treatment of ADHD in looked-after children (LAC).

**Recent findings:**

LAC are a very challenging population from a clinical and psychosocial standpoint, with higher mental health needs compared to non LAC. To date, no systematic review on the prevalence of ADHD, and its treatment, in LAC is available.

**Summary:**

We searched Pubmed, PsycInfo EMBASE + EMBASE CLASSIC, OVID Medline and Web of Science up to November 9 th, 2016. We found 24 papers meeting our criteria. The vast majority of the retained studies are from the USA and show rates of ADHD and of its pharmacological treatment substantially higher in LAC than those reported in national estimates. Future studies from countries other than the USA, aiming to understand the most cost-effective strategies, in the short as well as long term, to manage symptoms of ADHD in LAC are needed.

**Electronic supplementary material:**

The online version of this article (doi:10.1007/s40474-017-0116-z) contains supplementary material, which is available to authorized users.

## Introduction

Attention-deficit/hyperactivity disorder (ADHD) is characterized by impairing, persistent and pervasive levels of inattention and/or hyperactivity/impulsivity [1]. With an estimated worldwide prevalence of around 5% [2], ADHD is one of the most commonly diagnosed neurodevelopmental disorders. ADHD is frequently comorbid with other conditions such as oppositional defiant disorder, conduct disorder, specific learning disorders, mood and anxiety disorders, substance use disorders, sleep disturbances and other neurodevelopmental disorders such as autism spectrum disorder [3–8], as well as with somatic conditions such as obesity [6, 9, 10].

ADHD is a complex and heterogeneous disorder in terms of brain correlates, characterized by a dysfunctional interplay among several neuronal networks [11]. Its aetiology is accounted for by an interaction of genetic and environmental factors, the most common ones being prematurity, low birth weight and maternal smoking or alcohol during pregnancy [12].

Available treatments for ADHD include pharmacological and non-pharmacological interventions. Medications for ADHD comprise psychostimulant (e.g. methylphenidate and amphetamine derivatives) and non-psychostimulant drugs (e.g. atomoxetine, clonidine and guanfacine). A large body of evidence shows that ADHD medications are efficacious, at least in the short and medium terms, to control ADHD core symptoms [13, 14]. Non-pharmacological options for ADHD include, among others, parent training programmes, diet interventions, cognitive training and neurofeedback. Available evidence indicates that whilst the value of these interventions for ADHD core symptoms remains uncertain [15–18], they can effectively address associated conditions, such as oppositional behaviours in the case of parent training programmes [19].

Whilst ADHD has been extensively investigated in particular populations, such as preterm children or individuals in prisons, it has been neglected in others, such as looked-after children (LAC).

LAC are defined as those children who are being provided with substitute care [20]. The term “looked-after” is often used when referring to children who are in public care. Thus, LAC are inclusive of children in foster care, as well as children who are living with their parents but are subjected to care orders [21].

LAC are representative of a very challenging and complex population. Children in residential care settings are generally regarded as having greater mental health needs, in comparison to the general population of the same age [22]. According to House of Commons Education Committee [23], 42% of LAC aged between 5 and 10 years old in the UK were affected by a mental illness, as opposed to only 8% in the general population in that same age category. The study further noted that 49% of LAC aged between 11 and 15 were affected by a mental illness, with only 13.5% in the same age category in the general population. Additionally, 13.5% of children in care were using psychotropic medications; this figure stood three times higher in comparison to children living with their birth families [23]. Children entering foster care are generally in poor mental health not only as a result of risk factors such as parental mental illnesses or poverty, but also because of the fact that there is inadequate medical provision before entering into care.

Among the psychopathological risks that LAC are prone to, it has been reported that children in foster care are more prone to experiencing psychosocial (such as dysfunctional family dynamics) and biological risk factors (such as maternal smoking or use of alcohol during pregnancy) before and during their stay in care that makes them more susceptible to hyperactivity, impulsivity and inattention.

To our knowledge, literature on the prevalence and characteristics of ADHD, including its treatment, in LAC has not been systematically reviewed. Gaining insight into the prevalence of the diagnosis of ADHD in LAC, as well as of the rates of prescription of ADHD medication, is of relevance from a clinical as well as public health standpoint. The aim of this paper is to fill this gap, in order to provide relevant and updated information to patients, clinicians and managers when designing clinical pathways for the care of LAC.

## Methods

Methods were developed according to Preferred Reporting Items for Systematic Reviews and Meta-Analyses (PRISMA) [24] recommendations.

### Search Strategy

We searched the following electronic databases: PubMed, PsycInfo, Embase + Embase Classic, Ovid MEDLINE and Web of Sciences databases, with no language restrictions, from inception to November 9, 2016. The search terms and syntax for PubMed were as follows: (ADHD [tiab] OR Attention-deficit/hyperactivity disorder [tiab] OR attention deficit disorder with hyperactivity [tiab] OR Attention deficit [tiab] OR hyperkinetic disorder [tiab] OR hyperkinetic syndrome [tiab]) AND (“looked after children” [tiab] OR “foster care” [tiab] OR “residential setting” [tiab]). The search terms and syntax were adapted for the other databases and are reported in the Supplemental Material. We also searched bibliographic references from relevant papers.

We retained peer-reviewed, empirical quantitative studies providing information on the prevalence of ADHD, and/of its treatment, in LAC. We excluded case reports, case series, qualitative studies or non-peer-reviewed publications. As for the diagnosis of ADHD, we included studies based on a formal diagnosis of ADHD as per the Diagnostic and Statistical Manual of Mental Disorders (DSM), III, III-R, IV, IV-TR or 5 or the International Classification of Diseases (ICD), 10th edition, or studies where ADHD was defined based on scores above a cut-off on a validated ADHD questionnaire.

## Results

**Fig. 1:**
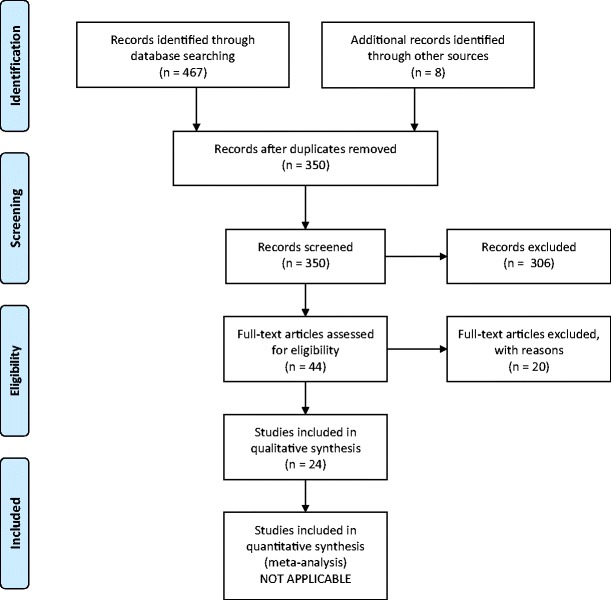
PRISMA flowchart illustrating study selection process

From an initial pool of 350 possibly relevant references, we retained 24 studies meeting our criteria (Supplemental Material). The details of the selection process are reported in Fig. 1, which shows the PRISMA flowchart. Table 1 summarizes the characteristics of studies reporting data on the prevalence of ADHD in LAC. Table 2 reports the characteristics of studies with data on the rates of medications to treat symptoms of ADHD in LAC. As shown in the tables, the vast majority of the studies retained in the systematic review were conducted in the USA and adopted a cross-sectional design. Sample sizes of LAC participants varied substantially across studies, from 87 to 51.500. Diagnostic procedures for ADHD were also heterogeneous across studies.

**Table 1:** Studies reporting data on the prevalence of ADHD in LAC

First author (year)	Country	Design	Sample size (*N* of youth in foster care)	Age (years)	Diagnosis of ADHD (tools and/or criteria)	Key findings
Bronsard (2011)	France	Cross sectional	183	13–17	DSM-III-R Diagnostic Interview Schedule for Children (DISC 2.25)	Prevalence of ADHD in LAC 3.8% (previous estimate of ADHD prevalence in the general population in France 2%).
Dos Reis (2001)	USA	Cross sectional	310	<19	ICD-9-CM	Comparison of 3 groups with ADHD: those in foster care, those receiving Supplemental Security Income (SSI) and those receiving other aids. Prevalence of ADHD in youth in foster care, those receiving Supplemental Security Income (SSI) and those receiving other aids 16, 7, 0.8%, respectively.
Dos Reis (2011)	USA	Cross sectional	2310 (1075 with ADHD)	<20	ICD-9 revision	Comparison of 3 groups with ADHD: those with foster care, those with low income and those with federally documented disability. Prevalence of ADHD diagnosis: foster care, 46.5%; disabled, 56%; and low income, 52%.
Garland (2001)	USA	Longitudinal	1618	16–18	DSM-III-R Computerized Diagnostic Interview Schedule for Children (C-DISC 2.25)	Prevalence of ADHD in child in welfare 21% (prevalence of with ADHD/disruptive behaviour disorder 38.7%)
Goodman (2004)	UK	Cross-sectional	1028	5–17	Prediction of diagnoses based on the Strength and Difficulties Questionnaire (SDQ)	68% of LAC detected as at risk for ADHD.
Harman (2000)	USA	Cross-sectional	3696	5–17	ICD-9-CM	Prevalence of ADHD in LAC, children in “Aid to Families with Dependent Children” and children in Supplemental Security Income 14.7, 3.9 and 19.8%, respectively.
Heneghan (2013)	USA	Longitudinal	815	12–15	Scores above cut-off in the Child Behavior Checklist (CBCL) and Youth Self-Report (YSR)	Prevalence of ADHD in LAC 18.5%.
Humphreys (2015)	Romania	Longitudinal Cohort	110	12	Diagnostic Interview Schedule for Children (4th edition; DISC-IV)	Children who had been institutionalized displayed higher rates of externalizing disorders such as ADHD (4.00 vs. 0.71 in comparison to those who were not).
Jee (2011)	USA	Cross sectional	138	11–17	Prediction of diagnoses based on the Strength and Difficulties Questionnaire (SDQ)	Risk for ADHD according to parents and youth 13 and 8%, respectively.
Lehmann (2013)	Norway	Cross sectional	279	6–12	Development and Well-Being Assessment (DAWBA) Development and Well-Being Assessment (DAWBA) DSM-IV criteria	Prevalence of ADHD in LAC 19%.
Linares (2010, 2013)	USA	Longitudinal	252	3–12	Diagnostic Interview Schedule for Children (4th edition; DISC-IV).	Prevalence of ADHD in LAC 55%.
McMillen (2005)	USA	Cross sectional	373	17	Diagnostic Interview Schedule for DSM-IV	Prevalence of ADHD in LAC 20%.
Raghavan (2008)	USA	Cross sectional	406	17	Diagnostic Interview Schedule IV (DIS 5)	Prevalence of ADHD in those leaving foster care 10.5%.
Vanderwerker (2014)	USA	Cross sectional	301.894	<18	ICD-9-CM	Prevalence of ADHD in LAC 17.3 vs. 6.5% in non-foster care youth.
Zito (2008)	USA	Cross sectional	472	0–19	ICD-9	Prevalence of clinician reported diagnosis of ADHD in LAC 38.8%.

Studies are listed in alphabetical order

**Table 2:** Studies providing data on the rates of medications for ADHD symptoms (and related symptoms) in LAC

First author (year)	Country	Design	Sample size (*N* of youth in foster care)	Age (years)	Diagnosis of ADHD (tools and/or criteria)	Key findings
Burcu (2014)	USA	Cross sectional	1338	2–17	ICD-9	Rates of atypical antipsychotic use in youth in foster care: more than threefold greater than those enrolled in income-eligible Medicaid categories.
Chen (2009)	USA	Cross sectional	4129	<21	ICD-9-CM	The rates of pharmacological treatment with ADHD medications was significantly higher in youth in foster care compared to those not in foster care (OR = 1.11, 1.02–1.20)
Dos Reis (2004)	USA	Cross sectional	87 with ADHD	<20	ICD-9	Comparison of 3 groups with ADHD: those in foster care, those with low income and those with federally documented disability. Rates of ADHD medication use: foster care, 81%; disabled, 88%; and low income, 83%.
Dos Reis (2011)	USA	Cross sectional	2310 (1075 with ADHD)	<20	ICD-9 revision	Comparison of 3 groups with ADHD: those with foster care, those with low income and those with federally documented disability Rates of ADHD medication use: foster care, 49.5%; disabled, 57.8%; and low income, 54.8%.
Dos Reis (2014)	USA	Retrospective	1491	≤6		Rates of ADHD medication prescription: age 3, <1%; age 4, 7%; age 5, 15%; and age 6, 22%.
Dos Reis (2001)	USA	Cross sectional	310	<19	ICD-9-CM	Comparison of 3 groups with ADHD: those in foster care, those receiving Supplemental Security Income (SSI) and those receiving other aids. Rates of psychostimulant use in youth in foster care, those receiving Supplemental Security Income (SSI) and those receiving other aids: 18, 5 and 1%, respectively
Ferguson (2006)	USA	Retrospective	473	<18	Not specified	Rates of stimulants use 56%.
Kamble (2015)	USA	Retrospective longitudinal analysis	9.448	6–17	ICD-9-CM	LAC had nearly twice the odds (OR = 1.83) of non-foster care children of receiving long-acting stimulants and second-generation antipsychotics concurrently.
Kreider (2014)	USA	Repeated cross-sectional design	51.500	6–18		Rates of stimulant use in 2004 and 2008, respectively, 21.1 and 22.8%.
Linares (2010; 2013)	USA	Longitudinal	252	3–12	Diagnostic Interview Schedule for Children (4th edition; DISC-IV).	Rate of stimulant use in LAC 38%.
Raghavan (2008)	USA	Cross sectional	406	17	Diagnostic Interview Schedule IV (DIS 5)	Rates of stimulant use in LAC 59%.
Zima (1999)	USA	Cross sectional	302	6–12	Child and Adolescent Functioning Assessment Scale (CAFAS), DSM-IV	Rate of psychostimulant use in the past year in LAC 62%
Zito (2008)	USA	Cross sectional	472	0–19	ICD-9	ADHD drugs (amphetamine, methylphenidate, others) in 55.9% of participants.

Studies are listed in alphabetical order

As for the prevalence of ADHD, only a minority of studies contrasted LAC and non-LAC, showing higher rates in LAC. The rest of the studies reported the prevalence of ADHD in LAC (without comparison to a non-LAC group), which was substantially higher than the national prevalence of ADHD in the study country [25]. Indeed, due to heterogeneity in the procedures to estimate ADHD rates, the prevalence of ADHD or risk for ADHD ranged from 3.8 to 68%. The rates of ADHD pharmacological treatment ranged from 22 to 81%. Likewise, across studies, the prevalence of ADHD pharmacological treatment was substantially higher than national estimates [26].

## Discussion

To our knowledge, this is the first systematic review aimed at comprehensively assessing the literature on the prevalence of ADHD, and its pharmacological treatment, in LAC.

Given the complex nature of the clinical presentation of LAC and their needs, some authors have reported concerns that ADHD might be under diagnosed in LAC, missing out on appropriate treatment [27]. However, the results of our systematic review would suggest that this is not the case, at least in the USA, where the vast majority of the studies retained in our systematic review were conducted. Nonetheless, the difference in the prevalence across studies is striking. In our view, such heterogeneity is accounted for by several factors. First, it should be noted that the tools and criteria for the assessment of ADHD varied across studies. More specifically, whilst some studies used a rigorous diagnosis according to formal ADHD criteria (as per DSM or ICD), other used a cut-off above a certain threshold on a validated ADHD questionnaire. Furthermore, the use of DSM or ICD may have introduced further heterogeneity, since the equivalent ICD diagnosis for ADHD (hyperkinetic syndrome) represents a more restricted category compared to the DSM ADHD. Beyond these important methodological factors, in our view, an additional source of heterogeneity across studies is represented by a possible discrepancy conceptualization of symptoms of hyperactivity, inattention and/or impulsivity in LAC. In particular, at least in some countries, these symptoms tend or at least tended to be considered as an expression of attachment disorder, rather than reflecting core symptoms of a primarily neurobiological or neurological disorder. In this regard, it is worthy to note that in the only study retained in our systematic review conducted in France [28], the prevalence of ADHD in LAC (3.8%) was clearly lower than that reported in the studies from the USA. It is interesting to note that child psychiatry and provision of child mental health in France have been strongly influenced by psychoanalytic models [29], which would contribute to practitioners formulate the symptoms of inattention, hyperactivity and impulsivity as being an expression of attachment issues, rather than of a primary ADHD. Indeed, the debate about the relationship between ADHD and attachment disorder is one of the most interesting ones in child psychopathology [30], but we believe also one of the less supported by evidence base. Whilst ADHD and attachment issues may be viewed as alternative constructs [31], it is also possible to conceptualize attachment issue as a risk factor contributing to ADHD [32].

Another concern is that ADHD is over diagnosed in LAC. This would particularly be favoured by misdiagnosing ADHD-like symptoms accounted for by disorders other than ADHD (e.g. anxiety, frequent in LAC) as “real” ADHD. In this regard, the use of the ICD category of hyperkinetic syndrome, which is ruled out in the presence of other disorders such as anxiety that can manifest with ADHD-like symptoms, would tend to reduce the risk of over diagnosis. However, rather than under or over diagnosis, in our view, the main issue is to which extent ADHD is correctly diagnosed in LAC, limiting false-positive as well as false-negative cases. Comparative studies across countries will be of interest in this respect.

Similarly to the prevalence of ADHD, the rate of ADHD medication prescription was general high across studies and higher than the national estimates of the use of ADHD drugs [26]. Data on the use of ADHD medication from each study should be interpreted with caution, since some of the studies reported lifetime prevalence, whilst others focused on actual prevalence. Furthermore, whilst some studies focused on psychostimulants or methylphenidate, others included several classes of ADHD medications (e.g. atomoxetine). Whilst over prescription is clearly a concern, in view of the potential side effects of ADHD medications (although in most cases, these are manageable), we believe that appropriate prescription is the key. Indeed, preliminary evidence shows the potential benefit of ADHD medications in LAC. A Danish study concluded that the decline in foster care caseloads in the period 1998–2010 would have been 45% smaller without a pharmacological treatment of ADHD [33].

Our work should be considered in the light of study strengths and limitations. Among the strengths, we highlight that we conducted a comprehensive search across a large set of databases and with no language/date restrictions. The main limitation is represented by the heterogeneity in the methodology across studies, which prevented us from conducting a formal meta-analysis based on the data retrieved in the studies retained in our systematic review.

In conclusion, this systematic review showed high prevalence of ADHD and high rates of ADHD pharmacological treatment in LAC, at least in the USA. Further methodologically sound research is needed from other countries. Perhaps more importantly, rather than assessing if ADHD is over or under diagnosed and treated in LAC, future research should aim to understand to which extent ADHD is appropriately diagnosed and treated in LAC and which are the most cost-effective strategies, in the short as well as long term, to manage symptoms of ADHD in LAC.

## Electronic Supplementary Material

ESM 1 (DOC 39 kb)
