# Supplementary material for: Attention-Deficit/Hyperactivity Disorder in Looked-After Children: a Systematic Review of the Literature
Source: Curr Dev Disord Rep. 2017 Jul 18;4(3):78–84. doi: 10.1007/s40474-017-0116-z (PMC5574951; doi:10.1007/s40474-017-0116-z)
Supplement: Supplementary file 1 — (DOC 39 kb) [file 40474_2017_116_MOESM1_ESM.doc]

**SUPPLEMENTAL MATERIAL**

**Search syntax and terms**

Pubmed

Search terms: (ADHD [tiab] OR Attention-deficit/hyperactivity disorder [tiab] OR attention deficit disorder with hyperactivity [tiab] OR Attention deficit [tiab] OR hyperkinetic disorder [tiab] OR hyperkinetic syndrome [tiab]) AND (“looked after children” [tiab] OR “foster care” [tiab] OR “residential setting” [tiab])

Results (9.11.16): 42 hits

PsycInfo+ EMBASE+EMBASE CLASSIC+OVIDMedline

(ADHD OR Attention deficit hyperactivity disorder OR attention deficit disorder with hyperactivity OR Attention deficit OR hyperkinetic disorder OR hyperkinetic syndrome) AND (looked after children OR foster care OR residential setting)

Results (9.11.16): 272 hits

WOS

ADHD OR Attention deficit hyperactivity disorder OR attention deficit disorder with hyperactivity OR Attention deficit OR hyperkinetic disorder OR hyperkinetic syndrome

looked after children OR foster care OR residential setting

Results (9.11.16): 190 hits

**TOTAL NUMBER OF REFERENCES TO SCREEN (after removing duplicates): 350**

**References of papers retained in the systematic review**

1. Bronsard G, Lancon C, Loundou A, Auquier P, Rufo M, Simeoni MC. Prevalence rate of DSM mental disorders among adolescents living in residential group homes of the French Child Welfare System. *Children and Youth Services Review.* Oct 2011;33(10):1886-1890.
2. Burcu M, Zito JM, Ibe A, Safer DJ. Atypical antipsychotic use among Medicaid-insured children and adolescents: duration, safety, and monitoring implications. *Journal of child and adolescent psychopharmacology.* Apr 2014;24(3):112-119.
3. Chen CY, Gerhard T, Winterstein AG. Determinants of initial pharmacological treatment for youths with attention-deficit/hyperactivity disorder. *Journal of child and adolescent psychopharmacology.* Apr 2009;19(2):187-195.
4. dosReis S, Owens PL, Puccia KB, Leaf PJ. Multimodal treatment for ADHD among youths in three Medicaid subgroups: disabled, foster care, and low income. *Psychiatric services (Washington, D.C.).* Sep 2004;55(9):1041-1048.
5. dosReis S, Tai MH, Goffman D, Lynch SE, Reeves G, Shaw T. Age-related trends in psychotropic medication use among very young children in foster care. *Psychiatric services (Washington, D.C.).* Dec 1 2014;65(12):1452-1457.
6. DosReis S, Yoon Y, Rubin DM, Riddle MA, Noll E, Rothbard A. Antipsychotic treatment among youth in foster care. *Pediatrics.* December 2011;128(6):e1459-e1466.
7. dosReis S, Zito JM, Safer DJ, Soeken KL. Mental health services for youths in foster care and disabled youths. *American journal of public health.* Jul 2001;91(7):1094-1099.
8. Ferguson DG, Glesener DC, Raschick M. Psychotropic drug use with European American and American Indian children in foster care. *Journal of child and adolescent psychopharmacology.* August 2006;16(4):474-481.
9. Garland AF, Hough RL, McCabe KM, Yeh M, Wood PA, Aarons GA. Prevalence of psychiatric disorders in youths across five sectors of care. *Journal of the American Academy of Child and Adolescent Psychiatry.* Apr 2001;40(4):409-418.
10. Goodman R, Ford T, Corbin T, Meltzer H. Using the Strengths and Difficulties Questionnaire (SDQ) multi-informant algorithm to screen looked-after children for psychiatric disorders. *European Child and Adolescent Psychiatry, Supplement.* 2004;13(2):II/25-II/31.
11. Harman JS, Childs GE, Kelleher KJ. Mental health care utilization and expenditures by children in foster care. *Archives of Pediatrics and Adolescent Medicine.* 2000;154(11):1114-1117.
12. Heneghan A, Stein REK, Hurlburt MS, et al. Mental health problems in teens investigated by U.S. child welfare agencies. *Journal of Adolescent Health.* May 2013;52(5):634-640.
13. Humphreys KL, Gleason MM, Drury SS, et al. Effects of institutional rearing and foster care on psychopathology at age 12 years in Romania: follow-up of an open, randomised controlled trial. *The lancet. Psychiatry.* Jul 2015;2(7):625-634.
14. Jee SH, Szilagyi M, Conn AM, et al. Validating office-based screening for psychosocial strengths and difficulties among youths in foster care. *Pediatrics.* May 2011;127(5):904-910.
15. Kamble P, Chen H, Johnson ML, Bhatara V, Aparasu RR. Concurrent use of stimulants and second-generation antipsychotics among children with ADHD enrolled in Medicaid. *Psychiatric services (Washington, D.C.).* Apr 1 2015;66(4):404-410.
16. Kreider AR, Matone M, Bellonci C, et al. Growth in the concurrent use of antipsychotics with other psychotropic medications in Medicaid-enrolled children. *Journal of the American Academy of Child and Adolescent Psychiatry.* Sep 2014;53(9):960-970 e962.
17. Lehmann S, Havik OE, Havik T, Heiervang ER. Mental disorders in foster children: A study of prevalence, comorbidity and risk factors. *Child and Adolescent Psychiatry and Mental Health.* 21 Nov 2013;7 (1) (no pagination)(39).
18. Linares LO, Li M, Shrout PE, et al. The course of inattention and hyperactivity/impulsivity symptoms after foster placement. *Pediatrics.* Mar 2010;125(3):e489-498.
19. Linares LO, Martinez-Martin N, Castellanos FX. Stimulant and atypical antipsychotic medications for children placed in foster homes. *PloS one.* 2013;8(1):e54152.
20. McMillen JC, Zima BT, Scott Jr LD, et al. Prevalence of psychiatric disorders among older youths in the foster care system. *Journal of the American Academy of Child and Adolescent Psychiatry.* January 2005;44(1):88-95.
21. Raghavan R, McMillen JC. Use of multiple psychotropic medications among adolescents aging out of foster care. *Psychiatric Services.* Sep 2008;59(9):1052-1055.
22. Vanderwerker L, Akincigil A, Olfson M, Gerhard T, Neese-Todd S, Crystal S. Foster care, externalizing disorders, and antipsychotic use among Medicaid-enrolled youths. *Psychiatric services (Washington, D.C.).* Oct 2014;65(10):1281-1284.
23. Zima BT, Bussing R, Crecelius GM, Kaufman A, Belin TR. Psychotropic medication treatment patterns among school-aged children in foster care. *Journal of Child & Adolescent Psychopharmacology.* 1999;9(3):135-147.
24. Zito JM, Safer DJ, Sai D, et al. Psychotropic medication patterns among youth in foster care. *Pediatrics.* Jan 2008;121(1):e157-163.
